# Identification of Attractants from Three Host Plants and How to Improve Attractiveness of Plant Volatiles for *Monochamus saltuarius*

**DOI:** 10.3390/plants13131732

**Published:** 2024-06-22

**Authors:** Yifan Dong, Dongping Chen, Siye Zhou, Zhengyi Mao, Jianting Fan

**Affiliations:** School of Forestry and Biotechnology, National Joint Local Engineering Laboratory for High-Efficient Preparation of Biopesticide, Zhejiang A & F University, Lin’an 311300, China; dyf@stu.zafu.edu.cn (Y.D.); klopq@foxmail.com (D.C.); z15857071498@163.com (S.Z.); 15382323773@163.com (Z.M.)

**Keywords:** *Monochamus saltuarius*, attractants, plant volatiles, aggregation pheromone

## Abstract

As a new vector insect of pine wood nematodes in China, the *Monochamus saltuarius* (Coleoptera: Cerambycidae) vectors pine wilt nematodes into healthy pine trees through feeding and oviposition, resulting in huge economic losses to forestry. A promising control strategy is to develop safe and efficient attractants. This study aims to screen for the key active volatiles of *Pinus koraiensis* (Pinales: Pinaceae), *Pinus tabuliformis* (Pinales: Pinaceae), and *Picea asperata* (Pinales: Pinaceae) that can attract *M. saltuarius*, and to study the synergistic attraction of the main attractant plant volatiles with ethanol and insect aggregation pheromones. The preference of *M. saltuarius* for three hosts is *P. koraiensis* > *P. tabuliformis* > *Picea asperata*. We detected 18 organic volatiles from three host plants. Through EAG assays and indoor Y-tube behavioral experiments, 3-carene, (-)-camphor, β-pinene, α-phellandrene, terpinolene, α-pinene, D-limonene, and myrcene were screened to have attractive effects on *M. saltuarius*. We found that 3-carene, β-pinene, and α-pinene are the most attractive kairomones in field experiments, which may play a crucial role in the host localization of *M. saltuarius*. Ethanol has a synergistic effect on the attractant activity of 3-carene and β-pinene, and the synergistic effect on β-pinene is the best. The mixture of ethanol, 2-undecyloxy-1-ethanol, and ipsdienol can significantly enhance the attraction effect of β-pinene on *M. saltuarius*. These new findings provide a theoretical basis for the development of attractants for adult *M. saltuarius* and contribute to the green control of *M. saltuarius*.

## 1. Introduction

*Monochamus saltuarius* (Coleoptera: Cerambycidae) is an important forest pest in Eurasia, feeding on important forest trees, such as *Pinus koraiensis* (Pinales: Pinaceae), *Pinus tabuliformis* (Pinales: Pinaceae), and *Picea asperata* (Pinales: Pinaceae) [1]. It affects tree growth and lumber quality and further reduces the economic value of wood. Recently, *M. saltuarius* has been identified as a vector for pine wood nematodes (PWNs) *Bursaphelenchus xylophilus* (Tylenchida: aphelenchidae) in Japan, Korea, and China, introducing PWNs into host plants through feeding and reproduction [2]. *B. xylophilus* is a plant-parasitic nematode native to North America that later invaded Europe and Asia and widely distributed in East Asia, causing deadly pine wood nematode disease and increasing the risk of tree death [3,4]. The spread of PWNs between host trees is mainly mediated by vector insects, and the *Monochamus* spp. are the main vectors [5,6,7,8,9]. In Northeast China, *M. saltuarius* plays a role in the transmission of pine wood nematodes and causes damage to important economic trees [8]. It is of great significance to develop attractants that can trap *M. saltuarius* efficiently for forest protection and management.

At present, the common strategies of insect pest control are chemical control, biological control, and breeding-resistant varieties [10,11,12,13]. Chemical control is the main method used to control pests, but there are also many problems with using chemical pesticide, such as polluting the environment, affecting non-target organisms, producing drug resistance, and so on [14,15,16,17]. It has been shown that insects determine the host location by detection of precise volatile ratios [18,19], and subtle changes in the volatile ratios of host plants can alter the behavior of insects and alter their perception and orientation [20]. Developing efficient attractants to trap *M. saltuarius* is a feasible and eco-friendly method for its controlling.

Plant volatiles can serve as semiochemicals to attract or repel insects. The volatiles of different host plants are significantly different, and are specific signal substances between plants in the same ecological environment, and are also important semiochemicals that directly affect the feeding and laying choice of adult insects. In short, plant volatiles play a very important role in the specific host and mate location for insects [21,22,23]. For example, the specific combination of α-pinene, camphene, and β-pinene was reported to be attractive to female *Conogethes punctiferalis* (Lepidoptera: Pyralidae) [24]. The compound 1-Octen-3-ol, a volatile from mango, induced the oviposition of female *Bactrocera dorsalis* Hendel (Diptera: Tephritidae) [25]. (Z)-3-Hexenyl-acetate in maize volatiles was reported to attract female *Spodoptera frugiperda* (Lepidoptera: Noctuidae) and stimulate oviposition [26]. 3-carene was found to be attractive for *Dendroctonus* and *Hylurgops* [27]. In addition, ethanol combined with host plant volatiles enhances the attraction of *Monochamus* species [28,29]. There are two types of volatile attractant pheromones in longhorn beetles (Coleoptera: Cerambycidae), aggregation pheromones and sex pheromones [30], which play an important role in pest control. In *Monochamus titillator* (Coleoptera: Cerambycidae) adults, males release aggregation pheromones and females release non-volatile contact pheromones [31], which enable male and female adults to recognize each other in sexual communication. The male adults of *Monochamus alternatus* (Coleoptera: Cerambycidae), *M. titillator*, and *M. saltuarius* lure female adults by releasing aggregation pheromones [32,33,34]. Moreover, pheromones of the same species in different subfamilies or genera have similar pheromone components [35]. The pheromones released by different species with the same host can also serve as mutualistic attractants for feeding or oviposition localization [33]. It is meaningful to find the key volatiles from the host plant and study the synergistic effect of plant volatiles and other pheromones for the development of *M. saltuarius* attractants.

*Pinus koraiensis*, *P. tabuliformis*, and *Picea asperata* are the main host plants of *M. saltuarius*. This article mainly analyzes the volatiles of these three host plants and selects host plant volatile organic compounds (VOCs) that have a seductive effect on *M. saltuarius*. At the same time, combining insect aggregation pheromones, the composite formulas of pheromones and VOCs were studied to screen attractants that can effectively attract adults of *M. saltuarius*. This plays an important role in green controlling the population of *M. saltuarius* and preventing the further spread of pine wilt disease.

## 2. Results

### 2.1. Feeding Preferences of M. saltuarius

**Figure 1:**
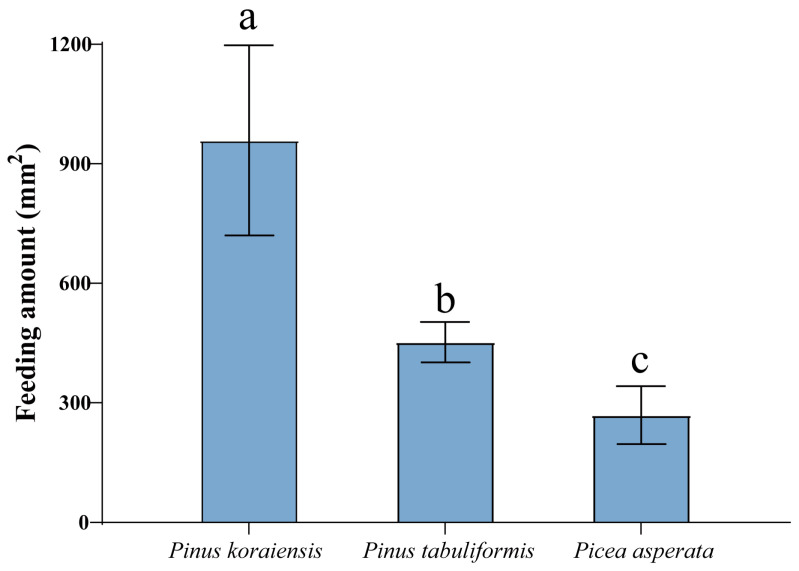
Feeding preference assay of *Monochamus saltuarius* to three host plants: *Pinus koraiensis*, *Pinus tabuliformis*, and *Picea asperata*. Different lowercase letters indicate significant differences at *p* < 0.05.

The feeding areas of three host plants were as follows: *P. koraiensis* (959.00 ± 238.73 mm^2^/6 head/3d) > *P. tabuliformis* (452.25 ± 50.79 mm^2^/6 head/3d) > *Picea asperata* (269.25 ± 72.59 mm^2^/6 head/3d). There were significant differences in the feeding amount of the three plants, and the adults of *M. saltuarius* ate more *P. koraiensis* than *P. tabuliformis* and *Picea asperata* (*p* < 0.05) (Figure 1).

### 2.2. Volatiles Identification of 3 Host Plants

There are mainly 18 volatile organic compounds in the volatiles of the three host plants of *P. koraiensis*, *P. tabuliformis*, and *P. asperata*, which can be divided into five categories: terpenes, aldehydes, ketones, alkanes, and esters (Table 1).

The volatile compounds of *P. koraiensis* contain four groups of compounds: terpenes (accounting for 98.83%), ketones (accounting for 0.74%), alkanes (accounting for 0.33%), and esters (accounting for 0.10%). Among the volatiles of *P. koraiensis*, terpenes make up the highest proportion, with D-limonene having the highest relative content of 28.00 ± 3.85%, followed by 3-carene, with a relative content of 22.75 ± 3.74%. The volatile compounds of *P. tabuliformis* contain two types of volatile compounds: terpenes (95.49%) and alkanes (4.51%), among which, β-pinene accounts for the largest proportion, at 34.62 ± 6.71%. There are three types of compounds in plant volatiles of *P. asperata*: terpenes (94.13%), aldehydes (1.99%), and ketones (3.87%). Among them, D-limonene makes up the highest relative content at 40.73 ± 8.14%, followed by 3-carene at 20.76 ± 6.18% (Table 1).

Limonene, α-pinene, and β-pinene were identified in three host plants, and their relative contents were high. The content of D-limonene in *P. asperata* was significantly higher than that of *P. tabuliformis* and *P. koraiensis* (*p* < 0.05). The contents of α-pinene and β-pinene in *P. tabuliformis* were significantly higher than those in *P. koraiensis* and *P. asperata* (*p* < 0.05). 3-carene, β-myrcene, and (-)-camphor were only present in *P. tabuliformis* and *P. asperata*, and the content of the three volatiles among the two plants was significantly different (*p* < 0.05). This may have contributed to the feeding preference of *M. saltuarius* (Table 1).

### 2.3. Electrophysiological Responses of M. saltuarius Adults to Volatiles in 3 Host Plants

**Figure 2:**
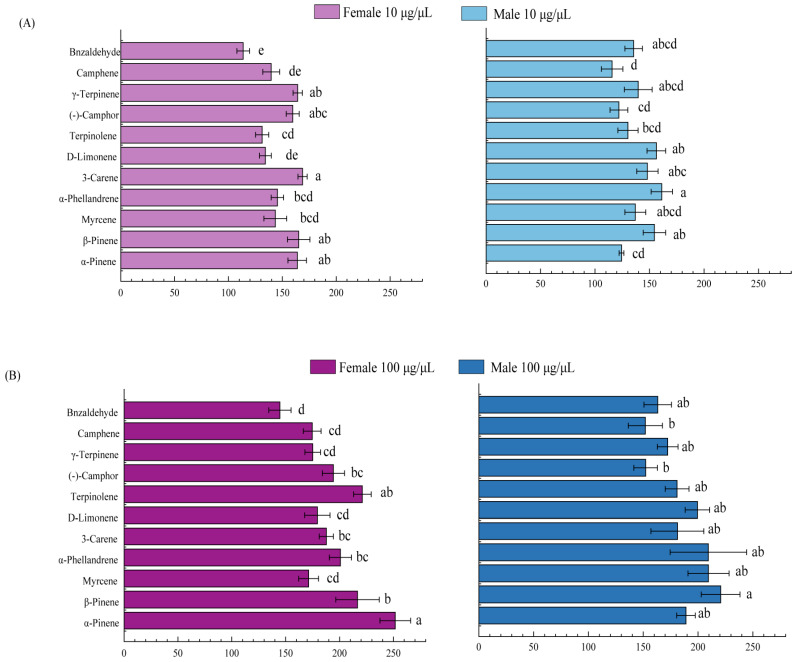
Electroantennography (EAG) relative response values of female and male *M. saltuarius* adults to the 11 active volatiles. (**A**) The 10 μg/μL active volatiles. (**B**) The 100 μg/μL active volatiles. Different lowercase letters on the bars are significantly different among EAG relative response values at different volatiles (*p* < 0.05 by Duncan’s multiple analysis).

From the analyzed plant volatiles, 11 compounds with a high relative content or those that have been reported to have attractant activity were selected for EAG experiments. As shown in Figure 2, the adults of *M. saltuarius* exhibited different degrees of EAG reaction to the 11 compounds, and the EAG relative response values of *M. saltuarius* to these volatiles at 100 μg/μL were greater than 10 μg/μL. At concentrations of 100 μg/μL, α-pinene, β-pinene, 3-carene, (-)-camphor, terpinolene, D-limonene, α-phellandrene, and myrcene, the relative antennal responses were 150% greater in both female and male insects (Figure 2).

### 2.4. Olfaction Selection Preference on Antennal-Active Host Volatiles by Y-Tube Assay

**Figure 3:**
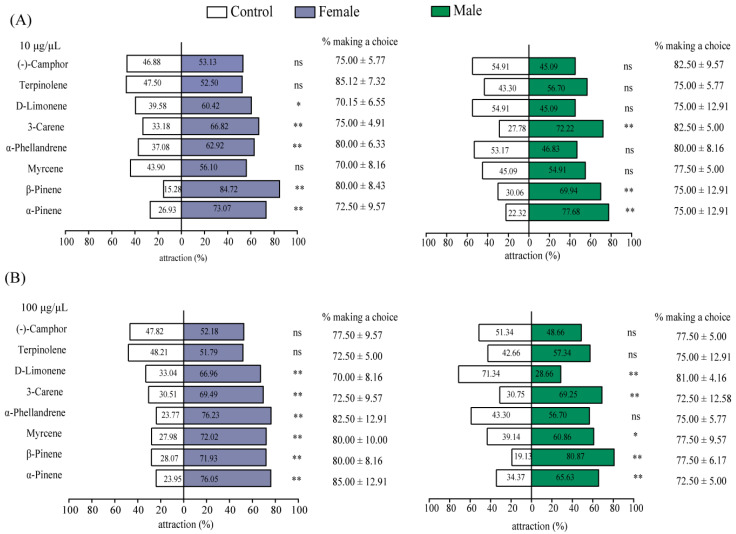
Olfactory behavioral responses of female and male *Monochamus saltuarius* adults to 8 antennal-active host volatiles at 10 μg/μL (**A**) and 100 μg/μL (**B**). Asterisks (** and *) in the figure indicate significant differences at the *p* < 0.01 and *p* < 0.05 levels, respectively, by the Chi-squared test. ‘ns’ indicates no significant difference.

To evaluate the effect of host plant volatiles on the directional behavior of M. saltuarius, eight host plant volatiles that can induce strong EAG responses were used for indoor Y-tube experiments. As shown in Figure 3, at the concentration of 10 μg/μL and 100 μg/μL, female insects can be significantly attracted by D-limonene (*p* < 0.05), but when the concentration of D-limonene is 100 μg/μL, female insects can be significantly attracted (*p* < 0.01) and male insects can be significantly repelled (*p* < 0.01). The 3-carene, β-pinene, and α-pinene at concentrations of 10 μg/μL and 100 μg/μL were highly attractive to both males and females (*p* < 0.01). α-Phellandrene at concentrations of 10 μg/μL and 100 μg/μL can significantly attract female adults (*p* < 0.01), but has no significant attractant or repellent effect on male adults (*p* > 0.05). When the concentration of myrcene was 10 μg/μL, it had no significant attractant or repellent effect on female and male *M. saltuarius* (*p* > 0.05), but when the concentration of myrcene was 100 μg/μL, it had a significant attractant effect on male (*p* < 0.05) and female (*p* < 0.01) insects. Camphor and terpinolene at concentrations of 10 μg/μL and 100 μg/μL had no significant attractant or repellent effects on female or adult insects (*p* > 0.05) (Figure 3). In conclusion, 3-carene, β-pinene, and α-pinene had the best trapping effect on M. saltuarius. At the same time, males and females showed different selectivity to D-limonene.

### 2.5. Forest Trapping Effects of 8 Active Volatiles to M. saltuarius Adults

**Figure 4:**
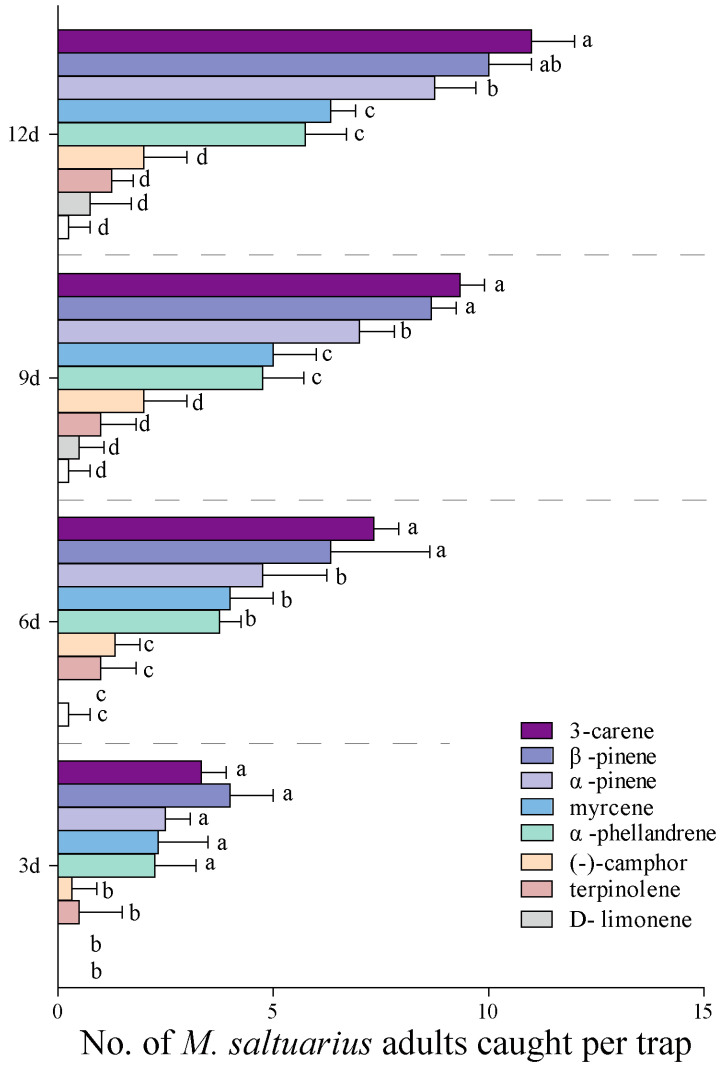
Effects of forest trapping of 8 antennally-active volatiles on *M. saltuarius* adults at 3 d, 6 d, 9 d, and 12 d. Different letters on the bars indicate significant differences among the active volatiles at the same conditions (*p* < 0.05, Duncan’s multiple analysis).

We tested the trapping effect of eight antennal active volatiles on *M. saltuarius* adults in the forest. It was found that different volatiles had different trapping effects on adults of *M. saltuarius*. As presented in Figure 4, during 12 days of continuous observation, camphor, terpinolene, and D-limonene had a poor trapping effect on *M. saltuarius* which was not significantly different from the control (*p* > 0.05). α-Phellandrene, myrcene, 3-carene, β-pinene, and α-pinene had a significant trapping effect on *M. saltuarius* that was significantly higher than that of the control (*p* < 0.05) at 6 d, 9 d, and 12 d (Figure 4). Moreover, the trapping effects of 3-carene, β-pinene, and α-pinene were significantly greater than α-phellandrene and myrcene at 9 d and 12 d (Figure 4).

### 2.6. Field Trapping Effects of Blends on M. saltuarius Adults

#### 2.6.1. Responses of *M. saltuarius* Adults to Blends of Single Volatile and Ethanol

**Figure 5:**
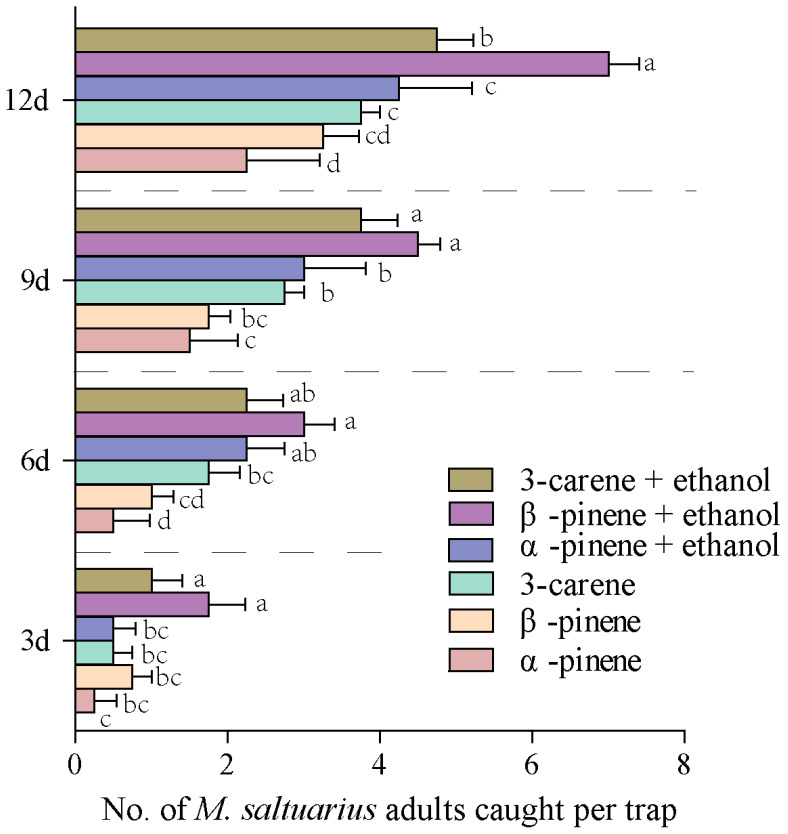
Mean catches of *Monochamus saltuarius* adults in traps baited from 3 d to 12 d, with 180 mL α-pinene, 180 mL β-pinene, 180 mL 3-carene, 135 mL α-pinene + 45 mL ethanol, 135 mL β-pinene + 45 mL ethanol, 135 mL 3-carene + 45 mL ethanol. Different lowercase letters on the bars mean significantly differences in the means by Duncan’s multiple analysis (*p* < 0.05).

We studied the synergistic effects of ethanol on 3-carene, β-pinene, and α-pinene, respectively. It was found that ethanol had a good synergistic effect. When the lures were used for 9 d and 12 d in the forest, ethanol had a significant synergistic effect on 3-carene, β-pinene, and α-pinene, and the synergistic effect on β-pinene was the best (Figure 5). The price of β-pinene is cheaper than 3-carene, and it is more suitable for the development of pheromone products. Therefore, the synergistic effect of 2-undecyloxy-1-ethanol, ipsenol, and ipsdienol on β-pinene was further studied.

#### 2.6.2. Responses of *M. saltuarius* Adults to Blends of β-Pinene, Ethanol and 2-Undecyloxy-1-ethanol

**Figure 6:**
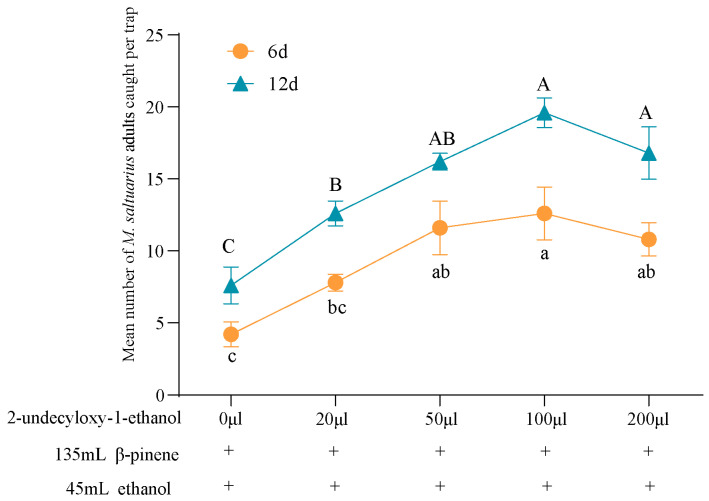
Mean catches of *M. saltuarius* adults in traps baited with 135 mL β-pinene, 45 mL ethanol, and different volumes of 2-undecyloxy-1-ethanol (0 μL, 20 μL, 50 μL, 100 μL, 200 μL). The data on the Y-axis are mean ± standard error (*p* < 0.05, Duncan’s multiple analysis).

Based on the synergistic effect of ethanol, we compared the synergistic effects of β-pinene and ethanol combined with different doses of 2-undecyloxy-1-ethanol on the attraction of *M. saltuarius* adults. It was found that β-pinene, ethanol, and 2-undecyloxy-1-ethanol had a synergistic effect. According to the trapping effect on day 6 and day 12, the number of trapped adults changed with the increase in the concentration of 2-undecyloxy-1-ethanol. The trapping efficacy was as follows: 0 μL < 20 μL < 50 μL < 200 μL < 100 μL 2-undecyloxy-1-ethanol (Figure 6). The trapping effect was best when 100 μL of 2-undecyloxy-1-ethanol was mixed with β-pinene and ethanol, and the mean number of adults captured was respectively, 12.6 ± 1.8 adults/6d/trap and 19.6 ± 1.0 adults/12d/trap, respectively (Figure 6).

#### 2.6.3. Responses of *M. saltuarius* Adults to Blends of β-Pinene, Ethanol, 2-Undecyloxy-1-ethanol, and Pheromones of Bark Beetles

**Figure 7:**
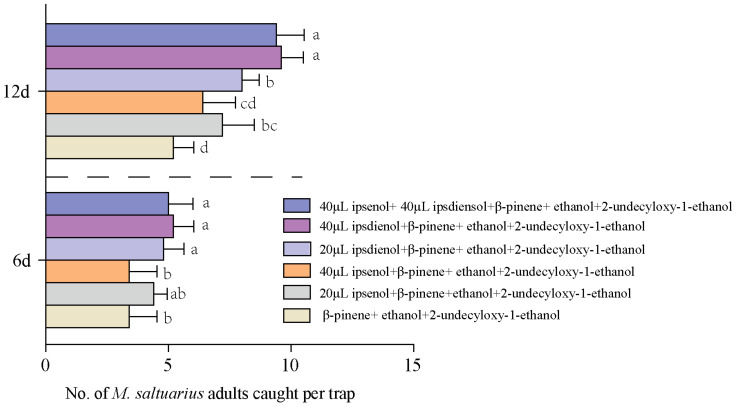
Mean number of *M. saltuarius* adults caught in traps baited with (1) 20 μL ipsdienol+ 135 mL β-pinene + 45 mL ethanol+ 100 μL 2-undecyloxy-1-ethanol, (2) 40 μL ipsdienol+ 135 mL β-pinene + 45 mL ethanol+ 100 μL 2-undecyloxy-1-ethanol, (3) 20 μL ipsenol+ 135 mL β-pinene + 45 mL ethanol+ 100 μL 2-undecyloxy-1-ethanol, (4) 40 μL ipsenol+ 135 mL β-pinene + 45 mL ethanol+ 100 μL 2-undecyloxy-1-ethanol, (5) 40 μL ipsdienol +40 μL ipsenol + 135 mL β-pinene + 45 mL ethanol+ 100 μL 2-undecyloxy-1-ethanol, (6) 135 mL β-pinene + 45 mL ethanol+ 100 μL 2-undecyloxy-1-ethanol. Different letters on the bars indicate significantly differences by Duncan’s multiple analysis (*p* < 0.05).

Ipsenol and ipsdienol are aggregation pheromones of bark beetles that have been reported to synergistically enhance attraction to some *Monochamus* species with plant volatiles and pheromones. Therefore, based on the above results, we compared the synergistic effects of ipsenol and ipsdienol on the mixture of 135 mL β-pinene, 45 mL ethanol, and 100 μL 2-undecyloxy-1-ethanol. It was found that, after the addition of 20 μL or 40 μL ipsdienol, the lure effect of the mixture on M. saltuarius was as follows: 40 μL ipsdienol > 40 μL ipsdienol+ 40 μL ipsenol > 20 μL ipsdienol > 20 μL ipsenol > 40 μL ipsenol (Figure 7). The addition of 40 μL ipsenol did not indicate a significant synergistic effect with the mixture of 135 mL β-pinene + 45 mL ethanol + 100 μL 2-undecyloxy-1-ethanol at 6 d and 12 d, while 20 μL ipsdienol and 40 μL ipsdienol showed a significant synergistic effect (Figure 7). Thus, ipsdienol and plant-derived compounds β-pinene, ethanol, and monochamol have a good synergistic effect, which is suitable for the development of efficient trapping agents for *M. saltuarius*.

## 3. Discussion

The selection and location of host plants are crucial for the survival and reproduction of herbivorous insects, which can distinguish nutritional and toxic compounds through their olfactory and gustatory systems [36,37,38]. Adults can detect and distinguish volatile substances from specific plants through VOCs [39,40,41,42], thus helping them search for host plants for oviposition or feeding.

This study collected and identified 18 VOCs from the following three host plants: *P. koraiensis*, *P. tabuliformis*, and *P. asperata*. These compounds mainly include terpenes, aldehydes, ketones, alkanes, and esters, with terpenes accounting for the largest proportion. α-Pinene β-pinene, and D-limonene were found in all three plants. Among terpenes, 3-carene and myrcene are present in *P. koraiensis* and *P. asperata*, while phellandrene and camphene are only identified in *P. koraiensis*. α-Pinene, β-pinene, 3-carene, myrcene, phellandrene, camphene, camphor, terpinolene, benzaldehyde, and γ-terpinene are relatively high in plants and some of them have been reported to attract other herbivorous insects [43,44]. D-Limonene is used as a repellent in some herbivorous insects. So, it is speculated that these 11 compounds may be potential attractants or repellent components for *M. saltuarius*. And, *M. saltuarius* revealed different degrees of EAG response to the 11 compounds, and the relative values of EAG responses to the 11 VOCs increase with increasing concentration. The EAG reaction cannot determine the selection or avoidance of compounds by *M. saltuarius*. From the analyzed compounds, 8 substances with strong EAG reaction values in *M. saltuarius* were selected for Y-tube behavior experiments. These results indicate that at two concentrations (10 μg/μL, 100 μg/μL), α- pinene, 3-carene, and β-pinene have significant attractant activity for both male and female adults of *M. saltuarius*, which may be the main plant-derived volatiles affecting host location.

The volatiles of the host plant of *M. saltuarius* are complex, and the composition and proportion of host volatiles may be influenced by various factors, such as plant age and physiological conditions, season, and biological and abiotic damage, all of which may affect the attractiveness to *M. saltuarius* [45,46]. Specific compounds from non-host plants may also be beneficial for host recognition [46,47,48,49,50]. This study indicates that, in forest trapping, 3-carene, β-pinene, and α-pinene have a strong attractive effect on *M. saltuarius*, with 3-carene having the highest number of individuals caught in traps.

Ethanol is a volatile indicator of stress and dying trees. When trees are stressed, the volatilization of ethanol from trees increases significantly. But, ethanol is attractive to some insects [51,52,53,54]. And, it has been reported that ethanol can work synergistically with α-pinene to increase the attraction effect of some beetles [49]. This study showed that β-pinene combined with ethanol had the best trapping effect on *M. saltuarius*. And, compared to using β-pinene, 3-carene, or α-pinene alone, the three volatiles combined with ethanol as a lure, have a higher number of individuals caught in traps. There is a synergistic effect between ethanol and the three compounds that enhances the attraction of *M. saltuarius*.

Compared with host volatiles, the aggregation pheromone of *M. saltuarius* is considered to have better attractant activity due to a higher sensitivity and selectivity [30,35]. The trapping effect of *A. glabripennis* on aggregation pheromones combined with plant volatiles was greater than that of aggregation pheromones alone [55]. Plant volatile compounds increase the attraction of sex pheromones to *Holotrichia parallela* (Coleoptera: Scarabaeidae), and a mixture of (E)-2-hexenyl acetate and sex pheromones results in significantly higher catches than sex pheromones alone [56]. It is reported that the combined use of different types of pheromones can effectively improve the attraction effect of *Xylotrechus pyrrhoderus* (Coleoptera: Cerambycidae), and when the sex pheromone (3-hydroxy-2-hexanone and 3-hydroxy-2-octanone) and the host pheromone (methyl benzoate) are mixed, its attraction effect is better [57]. The results showed that the aggregation pheromone 2-undecyloxy-1-ethanol had a synergistic effect on the combination of β-pinene and ethanol in the forest trapping. The trapping effect of the mixture with 2-undecyloxy-1-ethanol was significantly higher than that of the treatment without 2-undecyloxy-1-ethanol.

Different herbivorous insects may parasitize or lay eggs on the same host plant. To identify the same species on the host plant, more specific olfactory recognition pheromones are needed. As evolution progress, different insects feeding on the same host plant may also recognize each other’s pheromones. The suitable habitat of *M. saltuarius*, *Monochamus galloprovincialis* (Coleoptera: Cerambycidae), and bark beetles overlaps. The pheromones of bark beetles, ipsenol and ipsdienol, were also identified as synergists of the aggregation pheromone for *M. galloprovincialis* adults [58]. This study found that ipsdienol can also synergistically increase the trapping effect by mixing with aggregation pheromones of *M. saltuarius* and host plant volatiles.

This article describes a study on the feeding preferences of three hosts, the components of host volatiles, and the responses of *M. saltuarius* to host volatiles. The synergistic effects of host volatiles, ethanol, 2-undecyloxy-1-ethanol, and the pheromones of bark beetles were also evaluated. However, there are still some issues that need further exploration. The volatile compounds of host plants in the forest, as background odors, are usually complex in composition, and further exploration is needed to investigate the effects of the interactions between the components of host volatiles on the attraction of *M. saltuarius*. The different ratios of volatile organic compounds from different host plants on the attraction effect on *M. saltuarius* require further research. For other species with the same host, pheromones released by them can also serve as mutualists, which might be recognized by *M. saltuarius* for feeding or oviposition localization. Further research is needed to investigate the kairomonal attraction of pheromone components from other species to *M. saltuarius* or their interactions with identified host plant volatiles.

Developing attractants for longhorn beetles is a promising control strategy, but there are still some potential limitations and challenges. At present, most of the attractants developed can only trap sexually matured longhorn beetles, which have already spread pine nematodes through feeding and caused certain harm to trees. Therefore. it is urgent to develop attractants to trap longhorn beetles during their maturation feeding period. In addition, attractants were generally used to catch longhorn beetles to help control the pest population, but the control effects were limited. However, the attractants and chemical pesticides can be combined to improve the utilization efficiency of pesticides and reduce their use. In short, the development of attractants is promising, and we should make efforts to improve the effectiveness of attractants and overcome the challenges.

## 4. Materials and Methods

### 4.1. Insects and Plants

The insects of *M. saltuarius* used in the experiment were obtained from a farm in Qingyuan Manchu Autonomous County, Fushun City, Liaoning Province, China. They were lured and caught by traps, kept in insect cages, and fed with branches of *P. koraiensis*, and fresh branches were replaced every 2 days. Five-year-old healthy *P. koraiensis*, *P. tabuliformis*, and *P. asperata* were selected as test plants.

### 4.2. Chemicals

The CAS number, purity, and sources of chemicals involved in the research are listed in Table 2.

### 4.3. Host Plant Preference Assays of M. saltuarius

Different plants were used to study the feeding tendency of *M. saltuarius*. Host materials were obtained by cutting 30 cm long fresh branches from the tops of three types of plants: *P. koraiensis*, *P. tabuliformis*, and *P. asperata*. The cut branches were placed in insect cages (1 m × 1 m × 0.5 m), with a lighting setting of 16 L: 8 D. In each cage, 6 adults of *M. saltuarius*, 3 females and 3 males, who had been hungry for 6 h and were still active were used for host plant preference assays. We fed the adults of *M. saltuarius* for 3 d, replaced fresh branches daily, and calculated the feeding area of the branches. Each experiment was repeated 8 times. The feeding marks on the branches were replicated using sulfuric acid paper, and the feeding area of the branches was calculated using coordinate paper.

### 4.4. Volatile Analysis of Three Host Plants

The plant volatiles from 3 healthy host plants in healthy states were collected using the methods of Fan et al. [59]. The side branches of tested plants were sealed in oven bags (Polypropylene, 482 × 569 mm, Reynolds, Patrick County, VA, USA) for 6 h. We inserted two polytetrachloroethylene pipes were inserted into the bag opening and tied tightly with iron wire. The bag opening was wrapped and sealed with parafilm sealing film. One served as the exhaust rubber pipe and the other served as the intake rubber pipe. One end of the intake rubber tube was connected to an activated carbon sampling tube and the activated carbon tube was also connected to the air inlet of the atmospheric sampler through a polytetrachloroethylene tube. The outlet rubber tube was connected to a TENAX tube (which contains 200 mg of adsorbent), and the other end of the TENAX tube was also connected to the air inlet of the atmospheric sampler through a rubber tube, forming a closed loop that enabled air to flow out sequentially from the inlet of the atmospheric sampler, activated carbon tube, sampling bag, TENAX tube, and the outlet of the atmospheric sampler. The TENAX tube was activated at 300 °C for 3 h in advance as an adsorption tube. The atmospheric sampler had a flow rate of 0.5 L/min and, after 6 h of collection, the adsorption tube was removed and marked. The two ends were sealed tightly with parafilm sealing film and stored in a −80 °C freezer for testing. Volatile substances were analyzed using GC-MS-QP 2010 gas chromatography–mass spectrometry (Agilent, Santa Clara, CA, USA). Each experiment was repeated 3 times.

### 4.5. Electroantennography

For the electroantennography (EAG) assays, an EAG detector (Syntech-IDAC 4, Kirchzarten, Germany) was used to record the olfactory response of *M. saltuarius* to volatile compounds. We fixed the head was fixed and the complete antennae of the *M. saltuarius* was cut. The antenna was connected to the electrode using Spectra 360 conductive adhesive (Parker Laboratories, Fairfield, NJ, USA), with the base of the antenna connected to the reference electrode and the tip of the tail of the antenna connected to the recording electrode. The different chemicals were dissolved in paraffin oil at concentrations of 10 μg/μL and 100 μg/μL, respectively, and paraffin oil was used as control. An aliquot of 20 μL of solvent was applied to a 5 mm × 20 mm filter paper strip and the filter paper strip was placed completely inside the Pasteur pipette. The Pasteur pipette was connected to the airflow control device and the tip was inserted into the side hole of the mixing tube. A continuous airflow flow rate, set at 120 mL/min, stimulated the airflow flow rate to 20 mL/min, and the stimulation source was about 1.5 cm away from the antenna. The duration of each stimulation was 0.2 s, with a 1 min interval between the two stimuli to avoid antenna fatigue or adaptation. Paraffin oil was applied at the beginning and end of the trial (one antenna) to monitor antenna status. The EAG response values were normalized relative to the average response to paraffin oil. There were 8 replicates for each male and female adult.

### 4.6. Olfactory Behavioral Responses to Synthetic Active Volatiles

A total of 8 chemicals were selected from the volatiles of the main host plants of *M. saltuarius* for Y-tube olfactometer experiments. These compounds were as follows: (-)-camphor, terpinolene, D-limonene, 3-carene, α-phellandrene, myrcene, α-pinene, and β-pinene. The standards of each compound were prepared with n-hexane as solvent at concentrations of 10 μg/μL and 100 μg/μL, respectively, and n-hexane was used as the control. Specifications of Y-tube olfactometer were as follows: the inner diameter and length of main arm were, respectively, 9 cm and 40 cm, and the inner diameter and length of two lateral arms with a 60° angle at the Y-junction were 6 cm and 25 cm, respectively. Air was pushed through activated charcoal filter and a bottle with distilled water by a vacuum pressure pump (Beijing Municipal Institute of Labour Protection, Beijing, China). The air flow rate was about 500 mL/min. The adults of *M. saltuarius* were placed in a Y-shaped glass olfactometer to observe behaviors. One adult was taken each time for behavior selection measurement and observed for 10 min. For those who entered the arm with volatiles or the control arm for more than 1/3 of the distance and stayed for more than 15 s, they were recorded as *M. saltuarius* made a choice; otherwise, they were noted as unresponsive. After testing 5 insects, arm positions were switched to eliminate the influence of the experimental environment on the olfactory behavior of the spruce spotted beetle. A total of 40 males and 40 females were tested for each concentration of volatile matter. After each experiment, the experimental apparatus was cleaned with ethanol and dried.

### 4.7. Field Experiments

The experiments were conducted in Qingyuan Manchu Autonomous County, Fushun City, Liaoning Province, China (41°59′ N, 124°31′ E). Traps were hung in the sample plot, and the compounds were released through sustained-release bottles.

#### 4.7.1. Trapping Experiments of Single Compounds in Forests

(-)-Camphor, terpinolene, D-limonene, 3-carene, α-phellandrene, myrcene, α-pinene, and β-pinene were separately placed inside slow-release bottle at a volume of 180 mL. The slow-release bottles were used as lure core to put in traps. The empty slow-release bottles were used as a control. The 9 traps with different individual substance were put in a block with traps 20 m apart. A total of 5 blocks were set up for 5 repeated experiments. The interval between different blocks was 50 m. The survey was conducted every 3 days to count the adults of *M. saltuarius* caught by each lure core from 21 July to 1 August 2022, and then the adults in the trap collection cup were cleaned up. The lure core containing a single volatile was replaced by another trap during the same block every 3 days in order to reduce the impact of geographical location on the test results.

#### 4.7.2. The Synergies of Ethanol on the Trapping Effect of *M. saltuarius* in the Forest

Catches of *M. saltuarius* were compared in traps baited with 180 mL α-pinene, 180 mL β-pinene, 180 mL 3-carene, 135 mL α-pinene + 45 mL ethanol, 135 mL β-pinene + 45 mL ethanol, and 135 mL 3-carene + 45 mL ethanol. There were 5 replicates for each treatment. These assays were conducted from 14 to 23 May 2023, and the number of *M. saltuarius* was counted every 3 days.

#### 4.7.3. Trapping Assays on the Synergistic Effect of Aggregation Pheromones of *M. saltuarius*

The mixture combinations with aggregation pheromones for attracting *M. saltuarius* are shown in Table 3. These formulations were used to compare the synergistic effect of different concentrations of aggregation pheromones of *M. saltuarius*. The experiments were conducted from 29 May to 13 June 2023, and the number of captured adults of *M. saltuarius* was recorded every 6 days. Five replicates were carried out for each treatment.

#### 4.7.4. Field Attraction Tests to Explore Synergies of Bark Beetle Pheromones—Ipsdienol and Ipsenol

To explore the potential synergies of bark beetle pheromones, the formulations used to attract *M. saltuarius* in the forest were made as shown in Table 4. The survey was conducted from 12–24 July 2023, and the number of *M. saltuarius* caught by each trap was recorded every 6 days. Each treatment was repeated 5 times.

### 4.8. Data Analysis

All data were analyzed by SPSS 20.0 software, and the graphing was made by GraphPad Prism 9.5. The data from feeding experiments and forest experiments was conducted using one-way ANOVA and Duncan’s multiple comparisons. In the EAG experiment, a one-way ANOVA with Duncan’s multiple comparisons was also used to compare the relative EAG response values of male and female adults of *M. saltuarius* to different concentrations of different compounds. The indoor behavior data of Y-tube experiments were analyzed by using Chi-squared (χ^2^) goodness-of-fit tests.

## 5. Conclusions

In summary, we have identified plant volatiles that has attractive effects on *M. saltuarius*, identified key plant source volatiles for *M. saltuarius* host location, and further explored multi-component formulations using insect pheromones to optimize attractant formulations and improve trapping efficiency. We found that α-pinene, 3-carene, and β-pinene have significant attractant activity in adults of *M. saltuarius*, which may play an important role in the host location. β-Pinene, ethanol, 2-undecyloxy-1-ethanol, and ipsdienol had the best synergistic effect on luring *M. saltuarius*. This study provides a theoretical basis for controlling the population of *M. saltuarius* and preventing pine wood nematodes.

## Figures and Tables

**Table 1:** Volatiles identification of *Pinus koraiensis*, *Pinus tabuliformis*, and *Picea asperata*.

No.	Compounds	Category	No. CAS	Relative Content (%)		
				*Pinus koraiensis*	*Pinus tabuliformis*	*Picea asperata*
1	Nonane	Alkane	111-84-2	0.34 ± 0.05	0	0
2	α-Pinene	Terpenes	7785-26-4	11.41 ± 0.61 b	21.36 ± 5.61 a	9.49 ± 3.30 b
3	4-Carene	Terpenes	29050-33-7	1.36 ± 0.81 a	1.24 ± 0.21 a	0
4	Camphene	Terpenes	79-92-5	0.54 ± 0.04	0	0
5	Benzaldehyde	Aldehyde	100-52-7	0	0	2.02 ± 1.47
6	β-Pinene	Terpenes	18172-67-3	7.20 ± 0.65 b	34.62 ± 6.71 a	12.92 ± 3.49 b
7	β-Myrcene	Terpenes	123-35-3	22.59 ± 2.67 a	0	11.57 ± 2.89 b
8	α-Phellandrene	Terpenes	99-83-2	3.01 ± 0.53	0	0
10	β-Thujene	Terpenes	28634-89-1	0.24 ± 0.06	0	0
11	γ-Terpinene	Terpenes	99-85-4	4.68 ± 2.95 a	0.97 ± 0.39 a	0
12	β-Terpinene	Terpenes	99-84-3	0	7.57 ± 3.25	0
13	3-Carene	Terpenes	13466-78-9	22.75 ± 3.74 a	0	20.76 ± 6.18 b
14	D-Limonene	Terpenes	7705-14-8	28.00 ± 3.85 b	25.43 ± 6.46 b	40.73 ± 8.14 a
15	Terpinolene	Terpenes	586-62-9	0.54 ± 0.11 a	0.23 ± 0.09 a	0
16	Undecane	Alkane	1120-21-4	0	4.32 ± 0.14	0
17	(-)-Camphor	Ketones	464-48-2	0.77 ± 0.25 b	0	3.93 ± 1.60 a
18	Bornyl acetate	Ester	76-49-3	0.1 ± 0.06	0	0

Different lowercase letters indicate significant differences among different host plants (*p* < 0.05 by Duncan’s multiple analysis).

**Table 2:** Information of compounds in the study.

No.	Compounds	CAS	Purity	Sources
1	α-Pinene	7785-26-4	98%	J&K SCIENTIFIC
2	β-Pinene	18172-67-3	98%	J&K SCIENTIFIC
3	Myrcene	123-35-3	90%	J&K SCIENTIFIC
4	3-Carene	13466-78-9	90%	TCI AMERICA
5	α-Phellandrene	99-83-2	98%	J&K SCIENTIFIC
6	D-Limonene	5989-27-5	95%	J&K SCIENTIFIC
7	(-)-Camphor	464-48-2	96%	MACKLIN
8	Terpinolene	586-62-9	95%	J&K SCIENTIFIC
9	Camphene	79-92-5	78%	TCI AMERICA
10	γ-Terpinene	99-85-4	95%	J&K SCIENTIFIC
11	Benzaldehyde	100-52-7	98%	J&K SCIENTIFIC
12	Paraffin liquid	8012-95-1	99%	HUSHI

**Table 3:** Formulation of attracting *Monochamus saltuarius* with aggregation pheromone in forest.

Serial Number	Formulations
1	135 mL β-pinene + 45 mL ethanol
2	135 mL β-pinene + 45 mL ethanol + 20 μL 2-undecyloxy-1-ethanol
3	135 mL β-pinene + 45 mL ethanol + 50 μL 2-undecyloxy-1-ethanol
4	135 mL β-pinene + 45 mL ethanol + 100 μL 2-undecyloxy-1-ethanol
5	135 mL β-pinene + 45 mL ethanol + 200 μL 2-undecyloxy-1-ethanol

**Table 4:** Formulation of pheromones of bark beetles used to attract *Monochamus saltuarius* in the forest.

Serial Number	Formulations
1	135 mL α-pinene + 45 mL ethanol + 2-undecyloxy-1-ethanol
2	135 mL β-pinene + 45 mL ethanol + 2-undecyloxy-1-ethanol
3	135 mL β-pinene + 45 mL ethanol + 100 μL 2-undecyloxy-1-ethanol + 20 μL ipsdienol
4	135 mL β-pinene + 45 mL ethanol + 100 μL 2-undecyloxy-1-ethanol + 20 μL ipsenol
5	135 mL β-pinene + 45 mL ethanol + 100 μL 2-undecyloxy-1-ethanol + 40 μL ipsdienol
6	135 mL β-pinene + 45 mL ethanol + 100 μL 2-undecyloxy-1-ethanol + 40 μL ipsenol
7	135 mL β-pinene + 45 mL ethanol + 100 μL 2-undecyloxy-1-ethanol + 40 μL ipsenol + 40 μL ipsdienol

## Data Availability

All data are incorporated into the article.
